# The dynamics of the bacterial communities developed in maize silage

**DOI:** 10.1111/1751-7915.12751

**Published:** 2017-07-11

**Authors:** Javad Gharechahi, Zohreh Akhavan Kharazian, Sajjad Sarikhan, Gholamreza Salehi Jouzani, Mahnaz Aghdasi, Ghasem Hosseini Salekdeh

**Affiliations:** ^1^ Human Genetics Research Center Baqiyatallah University of Medical Science Tehran Iran; ^2^ Department of Biology Faculty of Science Golestan University Gorgan Iran; ^3^ Department of Systems Biology Agricultural Biotechnology Research Institute of Iran Agricultural Research, Education, and Extension Organization Karaj Iran; ^4^ Department of Microbial Biotechnology Agricultural Biotechnology Research Institute of Iran Agricultural Research, Education, and Extension Organization Karaj Iran

## Abstract

Ensilage provides an effective means of conserving summer‐grown green forage to supply as winter feed to ruminants. The fermentation process involved in the ensilage process relies on lactic acid bacteria (LAB). Here, 16S ribosomal DNA amplicon pyrosequencing was used to follow the dynamic behaviour of the LAB community during the ensilage of maize biomass, with a view to identify the key species involved in the process. The biomass used for ensilage was a single‐cross maize hybrid, harvested at the milk‐line stage. The crop was grown at three contrasting locations. Aspects of the physico‐chemical composition of the material and the LAB species present were sampled at 0, 3, 6, 14, 21 and 32 days after ensilage was initiated. In all three cases, members of the *Leuconostocaceae* family dominated the epiphytic bacterial community, notably *Leuconostoc* and *Weissella*, but some variation was noted in the abundance of certain *Leuconostocaceae* and *Lactobacillaceae* species, as well as that of some *Acetobacteraceae*,* Enterobacteriaceae* and *Moraxellaceae* species. The constellation of the microbiome which developed during the ensilage process differed markedly from that of the epiphytic one, with *Lactobacillaceae*, particularly *Lactobacillus* and *Pediococcus* spp. dominating. The abundance of heterofermentative *Leuconostocaceae* spp. in the epiphytic community and the extent of the transition from hetero‐ to homo‐fermentation during the initial ensilage period are important factors in determining silage quality.

## Introduction

In most temperate regions, green fodder is seldom available throughout the year. The two commonest means of conserving summer‐grown plant biomass to provide winter feed to ruminant animals during winter are drying to form hay and fermentation to form silage (Charmley, 2001). For the latter process, biomass is macerated at a crop growth stage chosen to maximize the dry matter (DM) content of the material (McDonald, 1991; Filya, 2004). The chopped plant materials are then compacted into large stacks or clamp silos using a roller or tractor, covered with a plastic sheet and carefully sealed to keep plant material away of atmospheric oxygen. Shortly after ensiling the residual oxygen present in the silo is consumed by plant cells and microbes associated with the ensiled material, and therefore, an anaerobic condition is quickly established. The material is then fermented anaerobically, largely through the activity of a range of lactic acid bacteria (LAB). The drop in pH of the silage which results from the production by LAB of lactic and acetic acid protects the silage from spoilage (McDonald, 1991; Weinberg and Muck, 1996).

Ensiling is a natural and spontaneous process that largely depends on anaerobic microbial fermentation. At the time of ensilage, the plant material harbours a range of microorganisms (the “epiphytic community”; Muck, 2013), but the fermentation process favours the multiplication of lactic acid bacteria (LAB; Ennahar *et al*., 2003; Yang *et al*., 2010; Eikmeyer *et al*., 2013; McGarvey *et al*., 2013) largely belonging to the genera *Lactobacillus*,* Pediococcus*,* Lactococcus*,* Enterococcus*,* Streptococcus* and *Leuconostoc* (McDonald, 1991; McGarvey *et al*., 2013; Muck, 2013). In some cases, the representation of LAB present does not effectively establish the required anaerobic and acidic conditions, leading to spoiling through the activity of other microorganisms (Woolford, 1990; McDonald, 1991; Muck, 2010). Attempts have been made to compensate for the low count of LAB in the epiphytic community and also to improve the fermentation efficiency, quality and aerobic stability of the silage by applying inoculant of laboratory‐expanded LAB to the ensiled plants (McDonald, 1991; Filya, 2003; Holzer *et al*., 2003; Filya *et al*., 2006; Muck, 2010; Eikmeyer *et al*., 2013). Thus, among a number of other factors, the constitution of the epiphytic community is an important determinant of silage quality. A knowledge of which LAB species are key to the production of healthy silage would aid the formulation of silage inoculants designed to bolster the natural epiphytic community.

The composition of the silage microbiome has been explored using various microbiological and DNA‐based techniques (Langston and Bouma, 1960; Ennahar *et al*., 2003; Parvin and Nishino, 2009; McEniry *et al*., 2010; Yang *et al*., 2010; McGarvey *et al*., 2013). In general, these approaches are not powerful enough to identify species present in low abundance, some of which may nevertheless be critical for optimal fermentation. However, the power of current DNA sequencing technologies now allows a much greater level of resolution than has been available in the past. For example, the microbial communities associated both with grass‐based silage inoculated with *Lactobacillus buchneri* (Eikmeyer *et al*., 2013) and with manyflower silvergrass supplemented with microalgae (Li *et al*., 2015) have been identified via the deployment of current sequencing technologies. The objective of the present study was to characterize the microbiome associated with ensiled maize biomass both before and during the ensilage process.

## Results

### Physico‐chemical analysis of the silage

**Figure 1:**
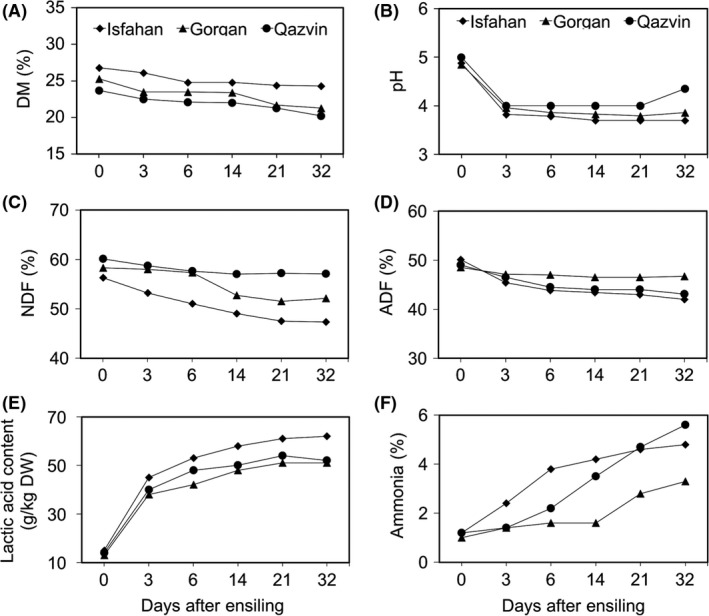
Characterization of physico‐chemical properties of the silages produced from maize biomass grown at three contrasting sites. The silages were sampled at 0, 3, 6, 14, 21 and 32 days post‐ensilage. Data are based on a dry matter content basis. DM, dry matter; NDF, neutral detergent fibre; ADF, acid detergent fibre.

At day 0 (prior to ensilage), the samples varied significantly (*P* < 0.006) with respect to DM: the biomass produced at the Isfahan site had the highest DM (26.8%) and that from the Qazvin site the lowest (23.7%; Fig. 1A). Over the initial ensiling period, the Isfahan samples retained their DM content more effectively than did those from either Qazvin or Gorgan; the latter had lost about 19% of their DM by day 32. Overall, silage samples collected from Gorgan displayed the same trend in DM reduction as that of Qazvin (Fig. 1A). The liquor pH of all the samples declined significantly (*P* < 0.0001), averaging 3.9 by day 3 and remaining relatively constant thereafter (with the exception of the Qazvin sample, which experienced a rise at day 32; Fig. 1B). With respect to NDF content, there was a significant difference between the Isfahan (56.3%) and Qazvin (60.1%) samples on day 0 (Fig. 1C). While the NDF content of the Qazvin samples remained relatively stable during the ensilage process (falling by just 4.8%), that of the Isfahan samples fell by 16.5% by day 32 (Fig. 1C). The ADF content also changed during the course of the ensilage process: the Gorgan samples were the least affected (−3.7%) and the Isfahan samples the most affected (−16.0%; Fig. 1D). The LA content of silages was sharply increased three days after the ensilage was initiated and continued to increase with storage time (Fig. 1E). The Isfahan samples showed the highest LA content compared to other two silage samples after 32 days of ensiling (*P* < 0.002). The ammonia content (averaging 1.1%) was comparable among the three sets of materials at day 0, and the effect of the ensilage process was to raise it significantly: for the Qazvin samples, the increase between days 0 and 32 was 4.6‐fold, while the increase for the Gorgan samples was more modest (3.3‐fold; Fig. 1F). The total OM content of silages samples was quite stable throughout the ensilage process (*P* < 0.059, Fig. S1). The overall conclusion was that among the three sites, the Isfahan‐grown material produced the highest quality silage.

### 16S ribosomal DNA amplicon sequencing

The pyrosequencing yielded 143 405 raw reads of mean length 404 nt. Around 65% of the sequences (93 263) was retained after the filtration step: this set had a somewhat higher mean read length (472 nt). The clustering algorithm sorted the sequences into 6459 OTUs, of which 227 were identified by the ChymeraSlayer algorithm as likely chimeric and so were discarded. Once the low‐abundance OTUs and those considered to reflect sequence artefacts had been removed from the analysis, 842 distinct OTUs (representing 78 756 sequences) remained for use in the subsequent diversity analysis. The details of number of reads generated per sample before and after quality filtering along with the number of OTUs detected in each sample before and after chimera detection and low abundant OTU filtering are presented in Table S1.

### Diversity analysis of the silage microbiome

A rarefaction analysis (Fig. S2) suggested that the sequencing depth achieved was sufficient to have captured the majority of the microbial diversity present in the materials both before and after ensilage. An estimation of species richness based on the Chao1 diversity index (Table 1) confirmed this conclusion. At day 0, the Isfahan and Gorgan samples harboured the fewest microbial species (Table 1). Even so, the estimated species richness present in these two samples indicated that the observed phylotypes covered over 80% of the microbial diversity. The Qazvin samples exhibited the greatest species richness both before and after ensilage, but the observed phylotypes only covered 73–83% of the diversity. Species richness tended to rise during the early period of ensilage (days 3–21), but had tailed off by day 32. The Shannon diversity parameter behaved in a similar manner, increasing up to day 3 and remaining fairly constant thereafter. At day 0, the Isfahan and Gorgan samples displayed the lowest Simpson indices (0.72 and 0.85, respectively) indicating a lower community evenness (Table 1). In contrast, the goods coverage, which estimates the proportion of the bacterial OTUs represented in each given sample, ranged from 0.95 to 0.99 (Table 1). The Isfahan and Gorgan samples at day 0 had a coverage of, respectively, 0.99 and 0.98, suggesting that a near complete set of the high abundance species had been sampled.

**Table 1:** Alpha diversity analysis of silages generated from maize biomass grown at three contrasting sites. Sampling was carried out both before and during ensilage. The indices report the number of phylotypes, along with the species richness, evenness and coverage

Sampling location	Sampling day	Observed species	Chao1	Shannon	Simpson	Goods coverage
Gorgan	0	114	137.0	4.05	0.85	0.98
	3	233	335.8	5.28	0.91	0.95
	6	261	340.1	5.74	0.94	0.95
	14	295	388.2	6.17	0.94	0.95
	21	285	374.7	6.15	0.95	0.95
	32	261	336.9	5.98	0.95	0.96
Isfahan	0	77	95.3	3.07	0.72	0.99
	3	185	242.9	5.35	0.92	0.97
	6	182	228.9	5.41	0.93	0.98
	14	185	215.2	5.38	0.93	0.98
	21	188	210.2	5.30	0.91	0.97
	32	190	222.7	5.43	0.93	0.97
Qazvin	0	228	311.2	5.89	0.95	0.96
	3	284	363.6	6.14	0.94	0.95
	6	276	339.4	6.14	0.94	0.96
	14	271	344.1	6.38	0.97	0.96
	21	280	340.6	6.40	0.97	0.96
	32	187	222.8	6.07	0.97	0.98

**Figure 2:**
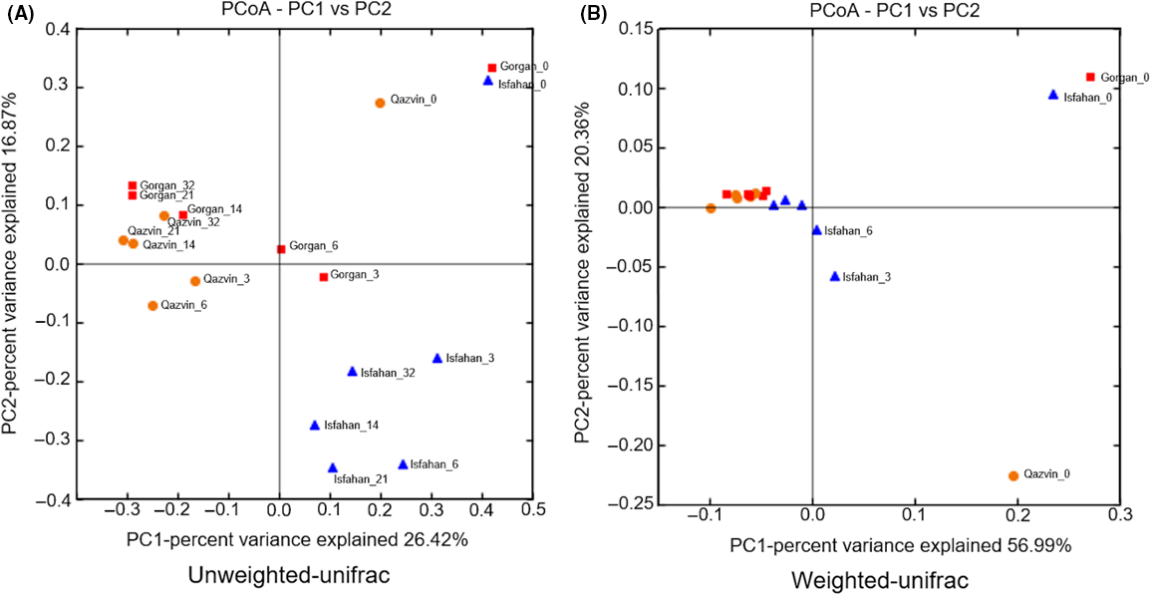
Beta diversity analysis. The PCoA plots presented report the phylogenetic separation between the various silages, taking into account either OTU presence/absence ((A) unweighted Unifrac metric) or OTU relative abundance ((B) weighted Unifrac metric). Only the first two components which explained higher variations are shown. Blue triangles represent Isfahan silage samples, red squares represent Gorgan silages, and orange circles represent Qazvin silages.

For the purposes of a beta diversity analysis, a calculation of pair‐wise similarities between the samples was made, using both weighted and unweighted Unifrac phylogenetic metrics; the results were visualized using PCoA plots (Fig. 2). The phylogenetic composition based on the unweighted Unifrac metric takes into account the presence and absence of OTUs: it was quite uniform across the three sampling locations at day 0 (Fig. 2A). The Isfahan origin ensiled biomass had a distinct microbial composition and appeared clustered in the PCoA plot. By day 14, the composition of the Gorgan and Qazvin samples became rather similar. Implementing the weighted Unifrac metrics, in contrast, showed that the composition of the ensiled material became uniform over time irrespective of the material's geographical origin (Fig. 2B). Based on this analysis, the composition of the Qazvin sample on day 0 was clearly different from that of the other two sites.

### Taxonomic composition of the silage microbial community

**Figure 3:**
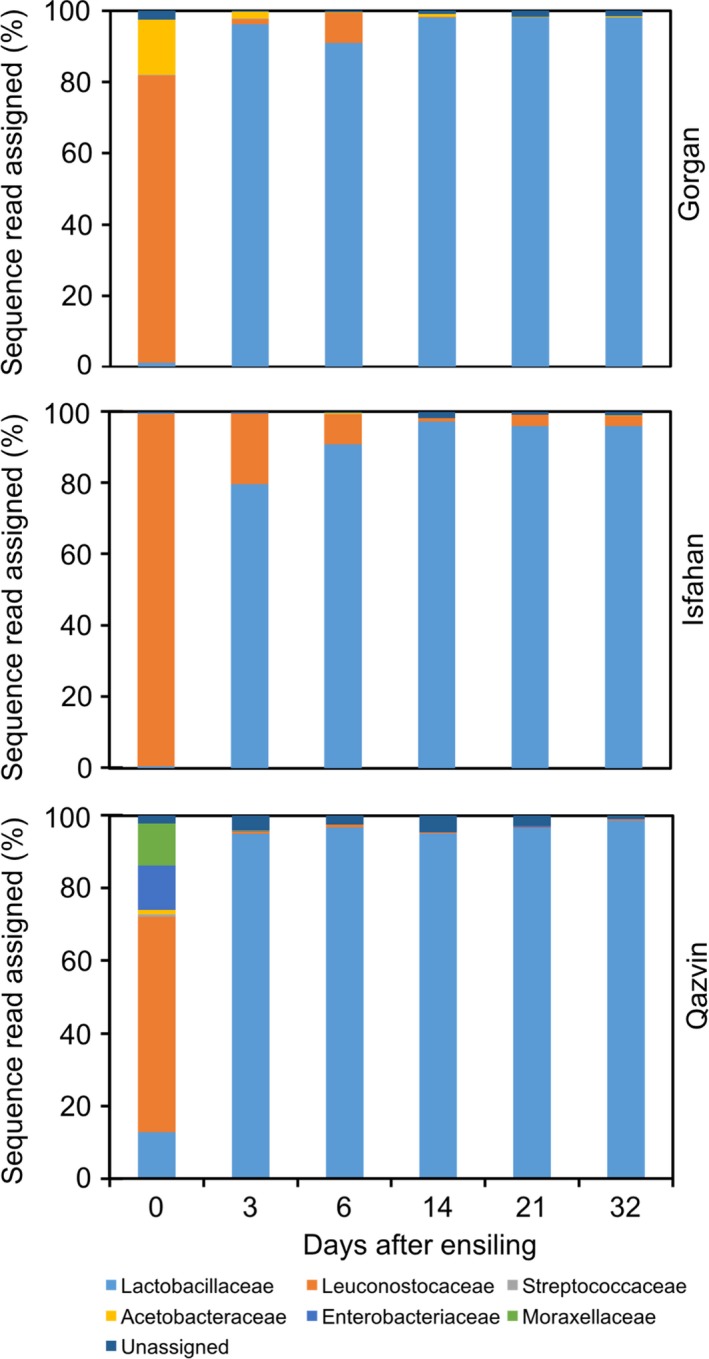
Composition of the microbial community in silages generated from maize biomass grown at three contrasting sites (Isfahan, Qazvin and Gorgan). Sampling was carried out both before (day 0) and during ensilage (days 3, 6, 14, 21 and 32). The stacked plots show the relative abundance of the specific phylotypes present in each sample on the basis of the percentage of reads assigned to corresponding OTUs.

Of the 842 OTUs identified, 752 (89.3%, 74 114 reads) were associated with *Firmicutes* species 46 (5.4%, 3344 reads) with *Proteobacteria* species, with the other 44 (5.2%, 1298 reads) remaining unannotated. At the family level, sequences were allocated to the *Lactobacillaceae* (71.7%), *Leuconostocaceae* (22.1%), *Acetobacteraceae* (2.5%), *Enterobacteriaceae* (0.9%), *Moraxellaceae* (0.8%) and *Streptococcaceae* (0.1%). The distribution of families on day 0 varied from site to site (Fig. 3). *Leuconostocaceae* species predominated, especially the genera *Leuconostoc* and *Weissella*. Site‐to‐site differences related for the most part to the relative abundance of *Lactobacillaceae*,* Acetobacteraceae*,* Enterobacteriaceae* and *Moraxellaceae*. The microbial community present at day 0 was heavily dominated by members of the *Leuconostocaceae* family, which accounted for an average across the samples of 78.8% of the species present. The Qazvin samples showed the greatest diversity at day 0, with *Leuconostocaceae* and *Lactobacillaceae* species accounting for, respectively, > 58% and > 12%, while both *Enterobacteriaceae* and *Moraxellceae* species accounted for > 10%; *Acetobacteraceae* and *Streptococcaceae* species were also represented, albeit at a low frequency. The Gorgan sample was dominated by the *Leuconostocaceae* (80%) and *Acetobacteraceae* (15%) and lacked any representation from *Moraxellaceae,* while the Isfahan material was overwhelmingly associated with *Leuconostocaceae* species (98.8%), with neither *Acetobacteraceae* nor *Moraxellaceae* species present (Fig. 3). The genera belonging to the *Lactobacillaceae* included both *Lactobacillus* and *Pediococcus*. Within the *Acetobacteraceae,* the genera present were *Acetobacter*,* Gluconobacter* and *Swaminathania*, with a number of reads not classifiable to genus. All the *Streptococcaceae* reads were assigned to the genus *Lactococcus*.

The taxonomic composition of the microbiomes changed markedly during the ensilage process. By day 3, the abundance of *Lactobacillaceae* had increased to 96.1% (Gorgan), 79.7% (Isfahan) and 95% (Qazvin), largely at the expense of *Leuconostocaceae* (Fig. 3 and Fig. S3); the abundance of the latter fell from 80.6% to 1.5% in the Gorgan silage, from 98.8% to 19.6% in the Isfahan silage and from 58.8% to 0.6% in the Qazvin silage. The abundance of both the *Acetobacteraceae* and *Enterobacteriaceae* was also diminished. At the genus level, most of the *Lactobacillaceae* representatives were assigned to *Lactobacillus,* with only a small representation of *Pediococcus* species (79 reads). In all samples, the abundance of *Lactobacillus* rose throughout the ensilage process. The commonest species present were *L. brevis* and *L. paralimentarius*, and only a minor number of OTUs were assigned to *L. plantarum* and *L. vaginalis*.

### RT‐PCR quantification of major LAB species contributing to the ensilage process

**Figure 4:**
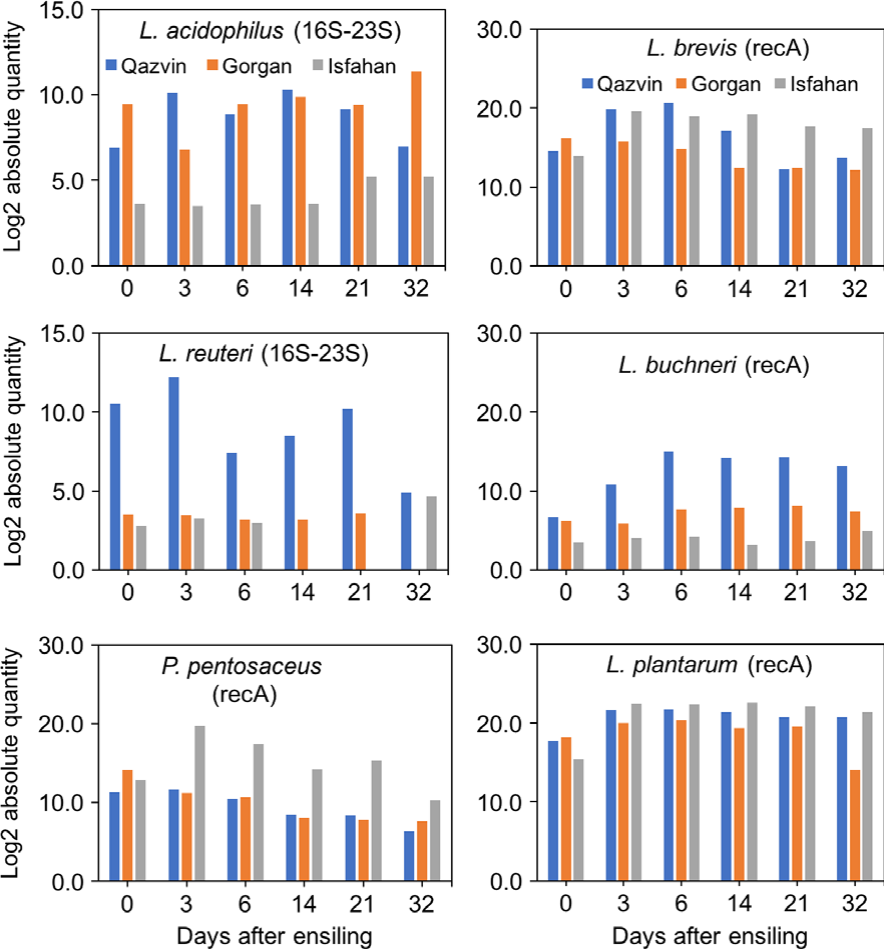
Real‐time PCR quantification of LAB contributing in the maize silage fermentation. RT‐PCR was run using species‐specific primers targeting intergenic spacer region of 16S‐23S rDNA and/or *recA* gene. For each species, a standard curve was constructed using 10‐fold serial dilution of plasmid containing the target PCR amplicon. Data are presented on the basis of log2 absolute count of bacteria detected in 2 ng of metagenomic DNA.

To further validate data obtained by amplicon pyrosequencing, we applied RT‐PCR to monitor the dynamics of some LAB species that are known as critical components of silage microbial community. For this analysis, primers were selected to target intergenic spacer region of 16S‐23S rRNA gene of *L. reuteri* and *L. acidophilus* and *recA* gene of *L. brevis*,* L. buchneri*,* L. plantarum* and *P*. *pentosaceus*. RT‐PCR analysis showed that the count of LAB such as *L. acidophilus*,* L. reuteri* and *L. buchneri* was significantly lower in the Isfahan silage compared to that in the Gorgan and Qazvin silages and likely have minor contribution in silage quality (Fig. 4). The Qazvin biomass that produced the lowest quality silage was associated with higher representation of *L. reuteri* and *L. buchneri* and significantly lower representation of *P*. *pentosaceus*. Interestingly, *P*. *pentosaceus* was detected at comparable level in all biomasses before ensiling (day 0), while significantly increased in abundance in the Isfahan silage following the ensilage process and decreased steadily in that of the other two sites with storage time (Fig. 4). RT‐PCR data also showed that *L. brevis*,* L. plantarum* and *P. pentosaceus* are among dominant LAB of maize epiphytic community.

### Predicted metabolic potential of silage metagenome

**Figure 5:**
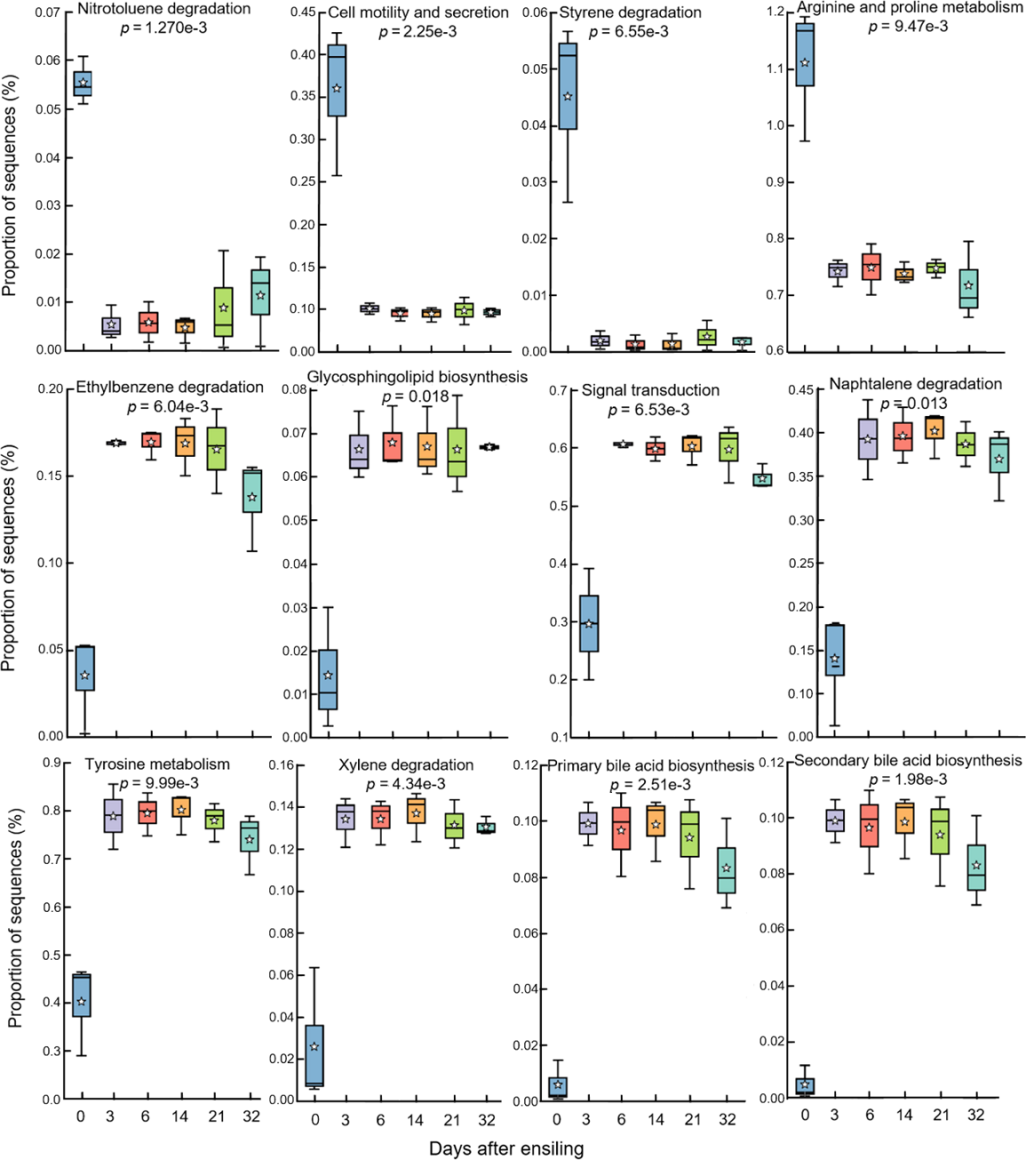
Functional prediction of maize silage metagenome using picrust (Langille *et al*., 2013). Box and whisker plots show the abundance of KEGG orthologs predicted to differ between the metagenome of maize biomass before (day 0) and 3, 6, 14, 21 and 32 days post‐ensilage. The functional metagenomes were calculated from OTUs picked against the greengenes database (version 13.5) using picrust. The pathway abundance values were estimated based on proportion of sequences assigned to each pathway and normalized to the total number of genes present in a particular pathway for each sampling day. Only significant KEGG pathways which were identified by One‐way ANOVA and Tukey's *post hoc* test using the stamp statistical package (Parks *et al*., 2014) are displayed. A *P*‐value < 0.05 was considered statistically significant.

PICRUSt enabled to identify major metabolic pathways associated with the ensilage process. The predicted metagenome of silage was annotated into 328 KO functional categories, in which many of them were not linked to the silage fermentation. The ensiling process was associated with depletion of pathways for nitrotoluene and styrene degradation, cell motility and secretion, and arginine and proline metabolism (Fig. 5). Importantly, ensiling was characterized by higher representation of pathways for ethylbenzene, naphthalene and xylene degradation as well as pathways for the biosynthesis of glycosphingolipid and bile acids. In addition, comparing metagenomes associated with maize biomasses obtained from different growing environments showed that the Isfahan silage had higher representation for propionate metabolism and lower representation for metabolism of xenobiotics, glycerophospholipid and cellular antigens (Fig. S4).

## Discussion

Differences in growing environment at the three sites were reflected in the contrasting levels of DM measured in the samples prior to ensilage. The DM content of the biomass strongly influences the ensilage process, because the LABs responsible for fermentation require moisture for their growth and reproduction (McDonald, 1991; Hu *et al*., 2009). Differences were also observed in the rate of DM loss during the ensilage process: silages in which DM loss is limited tend to be those where lactic acid fermentation is most effective (McDonald *et al*., 1973; McDonald, 1991). Silages which are initiated from biomass with a high water content generally develop a lower pH because of the higher concentration of water soluble sugars and more extensive fermentation (McDonald, 1991). Here, differences in the DM content had little discernible effect on the rate of decline in the pH during the ensilage period, which implied that the differences in DM content were not great enough to affect the temporal behaviour of the pH. The fermentation response of NDF and ADF suggested that the ensilage process improved the nutritional quality of the Isfahan silage much more than the other two silages. The amount of ammonia generated also varied: high levels may reflect excessive protein breakdown or amino acid de‐amination, and is usually associated with clostridial activity (McDonald, 1991; Driehuis and Oude Elferink, 2000). Overall, the indications were that in terms of silage quality (higher lactic acid concentration, lower pH and higher nutritional value), the biomass harvested at Isfahan was superior to the others.

With respect to the pre‐existing microbial community prior to the initiation of the ensilage process, the Isfahan material appeared to be less diverse than the other two (Table 1). The composition of the epiphytic community, as demonstrated by 16S ribosomal DNA sequencing, emphasized the dominance of *Leuconostocaceae* species, particularly the genera *Leuconostoc* and *Weissella*. The extent of this dominance (98% in the Isfahan biomass, 80% in the Gorgan biomass and 58% in the Qazvin biomass) appeared to be correlated with the final silage quality. *Leuconostoc* and *Weissella* are obligatory heterofermentative LAB species associated with numerous forage crops and silages (Cai *et al*., 1998; Ennahar *et al*., 2003; Yang *et al*., 2010). It is well established that these microbial species are critical for the normal fermentation and aerobic stability of silage (McDonald, 1991). The abundance of epiphytic LAB determines the adequacy of fermentation, contributes in silage quality and determines whether to apply bacterial inoculation to silage (McDonald, 1991; Lin *et al*., 1992b; Yang *et al*., 2010). *Pediococci*,* Leuconostoc*,* Streptococci* and *Enterococci* are all responsible for the lactate fermentation necessary to create the anaerobic acidic environment required for the growth and multiplication of LAB (Cai *et al*., 1998).

The variously sourced biomass was associated with a differential abundance of members of *Acetobacteraceae*,* Enterobacteriaceae*,* Lactobacillaceae* and *Moraxellaceae*. Two of these families (*Acetobacteraceae* and *Moraxellaceae*) were not represented in the Isfahan biomass, which produced the highest quality silage, and only harboured a limited abundance of *Lactobacillaceae* (0.49%) and *Enterobacteriaceae* (0.24%). In contrast, the Qazvin material, which produced the poorest quality silage, housed an abundance of *Erwinia* spp. (*Enterobacteriaceae*), *Lactobacillus* and *Pediococcus* spp. (*Lactobacillaceae*) and *Acinetobacter* spp. (*Moraxellaceae*). The latter are non‐LAB, aerobic, non‐fermenting, gram‐negative bacteria, sometimes associated not just with maize silage (Li and Nishino, 2011), but also form a component of the lettuce epiphytic community (Weiss *et al*., 2015). *Erwinia* spp., along with other gamma‐proteobacteria, colonize alfalfa (McGarvey *et al*., 2013), lettuce (Weiss *et al*., 2015), citrus (Yang *et al*., 2001) biomass, but are not generally associated with the ensilage process. Here, the abundance of *Enterobacteriaceae* fell significantly during the early period of the ensilage, thereafter remaining low, an observation which was taken to indicate their lack of importance for silage fermentation. *Acetobacteraceae* spp. were present in the Gorgan biomass at an appreciable abundance (15%), but not in that from the other two sites. Members of this family including *Gluconobacter oxydans* have been exploited as an additive to maize silage, as they encourage the development of silages with a reduced lactic to acetic acid ratio and a low ammonia concentration (Queiroz *et al*., 2013). The representation in the population of *Acetobacteraceae* was strongly decreased over the first three days of ensilage, suggesting that these microbes play at best a minor role in maize silage formation.

The ensilage process shifted the composition of the bacterial community from the heterofermentative LAB *Leuconostocaceae* to species belonging to the *Lactobacillaceae* which contained both homo‐ and heterofermentative microorganisms. The majority of the latter were classified to the genus *Lactobacillus,* with a few (< 1%) assigned to the genus *Pediococcus*. The shift from *Leuconostocaceae* to *Lactobacillaceae* LAB was incomplete in the Isfahan silage at day 3: the abundance of *Lactobacillaceae* increased from 0.49% at day 0 to 79% at day 3. In contrast, in the Gorgan and Qazvin silages, their representation was close to 100% at this time. The Isfahan silage showed the highest relative abundance of *Leuconostocaceae* (19.6%, 8.4%, 0.9%, 3% and 2.9% at, respectively, days 3, 6, 14, 21 and 32), which were uncommon in the other two silages. According to Li *et al*. (2015), *Lactobacillus* spp. quickly come to dominate in manyflower silvergrass silage, while McGarvey *et al*. (2013) have also observed a similar shift from gamma‐proteobacteria to homofermentative LAB in alfalfa silage. Similarly, the abundance of *Lactobacillus* spp. increases, while that of *Lactococcus* spp. decreases during grass ensilage (Eikmeyer *et al*., 2013). Obligate heterofermentative LAB are thought to be critical for biomass fermentation, especially in warmer climates (Muck, 2013); their role is to initiate fermentation by transforming soluble carbohydrates into lactic and acetic acid, thereby lowering the pH of the silage. As the pH falls, obligate heterofermentative LAB are disadvantaged, driving the shift towards homofermentative LAB (McDonald, 1991). The end‐point quality and nutritional value of silage are strongly dependent on a rapid establishment of both a low pH and a sufficiently anaerobic environment (Weinberg and Muck, 1996), which is promoted by an abundant representation of LAB in the epiphytic community. Here, we found that the abundance of heterofermentative LAB belonging to the family *Leuconostocaceae* is significantly higher in epiphytic community associated with the Isfahan silage (98.8%) and notably lower in that of the Qazvin silage (58.8%). Thus, the higher quality of the Isfahan silage can be partly attributed to the favourable constellation of its epiphytic community.

Heterofermentative LAB convert one mole of glucose into one mole of lactic acid, one mole of CO_2_ and a mole of either ethanol or acetic acid (Muck, 2010). Under anaerobic conditions, certain LAB have the ability to transform lactic acid to acetic acid (Elferink *et al*., 2001). The acetic acid produced by LAB is thought to inhibit the growth of yeasts, improving the silage's stability during storage and feeding, and minimizing nutrient loss during its long‐term storage (Moon, 1983; Hu *et al*., 2009; Li and Nishino, 2013). Most LAB used as inoculants for silage are homofermentative, producing two moles of lactic acid from one mole of glucose (Ennahar *et al*., 2003; Filya, 2003; Muck, 2010). At the species level, the LAB present in the various maize silages data indicate that *L. brevis* is one of the dominant LAB of maize epiphytic community, an observation that is in accordance with previous RT‐PCR data (Stevenson *et al*., 2006). Ensilage was associated with significantly higher prevalence of *L. brevis* during the initial days of ensiling (days 3 and 6) particularly in the Isfahan and Qazvin silages and remained at high level in the Isfahan silage and dropped to its initial level in the Qazvin silages thereafter (Fig. 4). These data suggest that the higher abundance of *L. brevis* is likely required for maintaining the quality of silages as the storage time of the ensiled material is extended. These results are somewhat in accord with previous data that indicate that *L. brevis* tends to populate later during the ensilage process (Lin *et al*., 1992a; Stevenson *et al*., 2006).

RT‐PCR showed that the abundance of homofermentative *L. plantarum* was quite high in the epiphytic community of maize biomass irrespective of the material's geographical origin and increased with the storage time particularly in the Isfahan silage. However, based on 16S rDNA sequencing data, OTUs affiliated to this species were present at a low abundance in the Isfahan and Gorgan silages, and this may indicate that the majority of OTUs belonging to this species skipped assignment at the species level. It has been reported that inoculating biomass with *L. plantarum* has a beneficial effect on silage quality (Cai *et al*., 1998; Hu *et al*., 2009). The presence of this species also appears to improve the aerobic stability of silage because it produces antifungal substances such as phenyl lactic and 4‐hydroxy‐phenyl lactic acids (Lavermicocca *et al*., 2000). The population of *L. plantarum* was positively correlated with the LA concentration (*r* = 0.5, *P *=* *0.03) and negatively with the pH (*r* = −0.5, *P *=* *0.02) and ADF content (*r* = −0.73, *P *=* *0.002), suggesting an important role for this bacterium in silage fermentation and fibre digestibility.

The prevalence of homofermentative *P. pentosaceus* in the epiphytic community of maize biomasses obtained from different growing location was quite comparable. However, ensiling was associated with a dramatic rise in its abundance in the Isfahan silage (peaked at day 3) but not in the other two silages, suggesting a key role for this species in efficient fermentation of maize biomass. A strong positive correlation was observed between the DM content of the ensiled material and the population of *P. pentosaceus* (*r* = 0.82, *P *=* *5.6e‐06), suggesting that the population of this species likely helped to maintain the DM content of the maize biomass during the ensilage process. *P. pentosaceus* is widely used in commercial inoculants which are applied to improve silage fermentation (McDonald, 1991). The results of RT‐PCR indicate that the population of *Lactobaccilli* such as *L. reuteri*,* L. acidophilus* and *L. buchneri* showed inverse correlation with the silage quality. Interestingly, *L. reuteri* showed a positive correlation with the NDF (*r* = 0.63, *P *=* *0.003) and the pH (*r* = 0.54, *P *=* *0.018) of the ensiled material, suggesting that this bacterium has a negative impact on maize silage fermentation. *L. buchneri* is a heterofementative LAB that is frequently used as an inoculant for improving aerobic stability of silage (Holzer *et al*., 2003; Hu *et al*., 2009; Eikmeyer *et al*., 2013). Inoculation of grasses with *L. buchneri* is known to result in higher concentration of acetic acid, lower concentration of lactic acid, higher pH and higher concentration of alcohols such propanediol and ethanol (Hu *et al*., 2009; Eikmeyer *et al*., 2013). Here we found that the population of *L. buchneri* has a strong negative correlation with the DM content of the ensiled material (*r* = −0.85, *P *=* *8.7e‐07), suggesting that the presence of this species is likely associated with higher DM loss. This finding is in accord with the previous study that showed that DM loss increases with increasing the inoculation level of *L. buchneri* above 10^5^ cfu g^−1^ (Driehuis *et al*., 1999). This DM loss could be explained in part by the mode of fermentation performed by this bacterium, which is a heterofermentative LAB. In heterofermentative fermentation for every molecule of acetic acid formed, an equivalent molecule of carbon dioxide is generated (McDonald, 1991); therefore, a considerable loss of DM would be expected with heterofementative fermentation (Wilkinson and Davies, 2013).

The pattern of gene abundance in the microbial community of maize biomass obtained from different growing sites, as predicted by PICRUSt, suggested the enrichment of genes for propionate metabolism in the Isfahan silage. Propionic acid is widely used as an additive to the ensiled material to restrict the growth of moulds and yeast (Oladosu *et al*., 2016). The high concentration of propionate is known to associate with improved aerobic stability, less spoilage and limited mycotoxin formation in corn silage (Kung *et al*., 1998). These data suggest a potential link between propionate metabolism and silage quality. Moreover, functional metagenome prediction highlighted that the Isfahan silage was also benefited from less representation of pathways for metabolism of xenobiotics and glycerophospholipid.

Comparing the predicted metagenome of maize biomass before and after ensiling demonstrated the higher representation of pathways for degradation of toxic compounds such as ethylbenzene, xylene and naphthalene in the silage community, suggesting that degradation of toxic compounds could be an important function of microbes involved in silage fermentation. Ensiling also led to an increased metabolism of primary and secondary bile acids, suggesting a potential link between bile acid metabolism and silage fermentation. In human, bile acids appear to play a critical role in regulating gut microbiota composition (Kakiyama *et al*., 2013; Ridlon *et al*., 2014). Diet supplementation of cholic acid, a bile acid, to rats showed that it has a favoured effect on the population of *Firmicutes* and a negative impact on the population of *Bacteroidetes* (Islam *et al*., 2011). These data suggest that bile acids may be involved in silage fermentation likely through facilitating the expansion of LAB, which are Gram‐positive bacteria belonging to the phylum *Firmicutes*. The role of bile acid metabolism in silage fermentation deserves further analysis.

It's worth to note that the functional metagenomes predicted by PICRUSt should be interpreted with caution as they were predicted from genomic sequences which might be differ from genetic constituent of microbial community in question. In addition, during closed‐reference‐based OTU picking which is required for PICRUSt analysis a significant proportion of reads could not be taxonomy assigned and therefore will be ignored from metagenome prediction. Many sequences can only be assigned at the genus level or even at higher taxonomic ranks making metagenome prediction unreliable. Bias in amplification of 16S rDNA might also lead to an incomplete picture of community structure. All these limitations might lead to an inaccurate estimation of functional metagenomes.

## Conclusion

In summary, the constitution of the epiphytic microbial community associated with maize biomass is influenced by the growing conditions obtaining during the production of the biomass. It is dominated by heterofermentative LAB belonging to the family *Leuconostocaceae*, in particular *Weissella* and *Leuconostoc*. After ensilage was initiated, the abundance of *Leuconostocaceae* fell sharply, while that of the *Lactobacillaceae* (*Lactobacillus* and *Pediococcus*) increased. The Isfahan silage which was characterized by a higher initial DM, a lower epiphytic microbial diversity, a higher pre‐ensilage abundance of *Leuconostocaceae* produced the highest quality silage among the three growing sites sampled. Further RT‐PCR analysis demonstrated that the Isfahan silage likely benefited from higher representation of *P. pentosaceus*,* L. brevis* and *L. plantarum* and lower representation of *L. buchneri*. Functional metagenome prediction also showed an association between the ensilage process and enrichment of pathways for propionate and bile acid metabolism and those for degradation of toxic compounds such as ethylbenzene, xylene and naphthalene. These results suggest that silage microbiome may play a key role in detoxification of plant‐derived toxic metabolites.

## Experimental procedures

### Plant material and ensilage

The biomass used for ensilage was obtained from the whole plant of a single‐cross maize hybrid (hybrid 704). A crop of this hybrid was raised in the field at three contrasting sites in Iran: Gorgan (temperate), Isfahan (warm and dry) and Qazvin (cold and dry). Plants were harvested at the milk‐line kernel maturity stage and chopped to a length of 2–4 cm using a conventional field chopper. The macerated material was packed firmly into a 10 × 50 cm polyethylene laboratory‐scale silo, which was sealed with an airtight lid and stored at ambient temperature. The vessels were equipped with a valve to allow the draining of excess liquid and the release of accumulated gas: they were opened every other day. Samples were taken from each silo at 0, 3, 6, 14, 21 and 32 days post‐ensilage. Each cropping site was represented by three silos.

### Physico‐chemical analysis of the silage

The following parameters were assessed for each sample of ensiled material: % neutral detergent fibre (NDF), % acid detergent fibre (ADF), lactic acid (LA) content, % organic matter (OM) and DM, as well as the pH and ammonia content of the liquor. DM was determined following a 48 h baking of the sample in a fan‐assisted oven running at 60°C. The dried material was ground finely enough to pass through a 1 mm sieve, and the powder was assessed for its NDF and ADF content using, respectively, a sulfite‐ and an amylase‐based assay, according to protocols described by Van Soest *et al*. (1991). To determine the silage liquor's pH, a 20 g sample was homogenized in 180 ml of distilled water for 5 min and the pH measured directly. To determine its ammonia content, an aliquot of the same aqueous extract was filtered through Whatman No 1 paper (Whatman, Maidstone, UK) and then acidified by adding H_2_SO_4_ following the protocol described (Weatherburn, 1967). LA content was measured in cold water extract of the silages via spectrophotometry approach as described by Barker and Summerson (1941).

### Microbial cell recovery and DNA extraction

Ten grams of silage samples per silo was homogenized in 30 ml of dissociation buffer (100 mM of Tris‐HCl, 50 mM of EDTA, 150 mM of NaCl, pH 8.0) for 30 min. The mixture was vigorously mixed every 5 min and then passed through a mesh filter. The filtrate was collected in a sterile container and centrifuged at 8,000 ×  *g* for 10 min at 4°C. The resulting pellet was washed twice in the same buffer and re‐centrifuged (8,000 ×  *g*, 10 min, 4°C), and the pellet was resuspended in 1.4 ml of stool lysis buffer (ASL buffer), a component of the QiaAmp^®^DNA Stool Mini kit (Qiagen, Valencia, CA, USA). DNA was extracted according to the manufacturer's protocol for isolating DNA from stools for pathogen detection.

### PCR amplification

The V1‐V3 hypervariable region of the 16S ribosomal RNA was PCR‐amplified using the universal primer pair 27F (5′‐AGAGTTTGATCCTGGCTCAG)/534R (5′‐ATTACCGCGGCTGCTGG), which yields a ~500 bp amplicon suitable for pyrosequencing using Roche 454 FLX titanium chemistry. To barcode the PCR products, a unique multiple identifier 10 nt sequence was added to the 5′ end of the 534R primer followed by the 454 A‐key primer sequence (5′‐CCATCTCATCCCTGCGTGTCTCCGACTCAG). The B‐key primer sequence (5′‐CCTATCCCCTGTGTGCCTTGGCAGTCTCAG) was appended to the 5′ end of 27F. PCRs (50 μl) were run in triplicate: each reaction comprised 25 μl of PCR master mix (Thermo Fisher Scientific, www.thermofisher.com), 0.5 μM of each primer, 30 ng of microbial DNA and 22 μl of double‐distilled water. The PCR regime consisted of an initial 94°C/5 min denaturation, followed by 30 cycles of 94°C/30 s, 56°C/40 s, 72°C/30 s, and finished with a 72°C/7 min extension. Following their electrophoresis, the amplicons were recovered from an agarose gel using a Qiaquick^®^Gel Extraction kit (Qiagen), quantified fluorometrically and pooled in equimolar fashion before being subjected to pyrosequencing on a 454 GS FLX titanium sequencer (Roche, www.roche.com) based at the Beijing Genome Institute (www.bgi.com). The raw sequence data have been deposited in the NCBI short read archive (SRA).

### Sequence analysis

The 16S rDNA gene sequences were analysed using the qiime pipeline version 1.9.1 (Caporaso *et al*., 2010b), as described previously with minor modification (Gharechahi *et al*., 2015). Sequences shorter than 200 nt and longer than 1000 nt were discarded, along with those which contained ambiguous bases, those having a mean quality score < 25, those containing runs of six or more of the same base, those containing a missing qual score and those including > 2 mismatches from the primer sequences. The remaining sequences were then clustered, using uclust software (Edgar, 2010), into operational taxonomic units (OTUs), based on a criterion of > 97% sequence similarity. The most abundant sequence in each OTU was selected as being representative, and these sequences were then aligned against the greengenes core set (gg_13_8) (DeSantis *et al*., 2006), using the PyNAST aligner set with a minimum sequence identity of 75% (Caporaso *et al*., 2010a). Taxonomies were assigned to each OTU using the Ribosomal Database Project naïve Bayesian classifier (Wang *et al*., 2007), applying a minimum confidence value of 0.8. The alignments were filtered to remove gaps and hypervariable regions and to define conserved and non‐conserved positions using a Lane mask (Caporaso *et al*., 2010a). A phylogenetic tree representing the relationship between OTUs was constructed from the filtered alignment using fasttree software (Price *et al*., 2009). Chimeric OTUs were detected using chimeraslayer (Haas *et al*., 2011) and removed from the OTU table. Low‐abundance OTUs (representing < 0.01% of the sequences) were discarded. To avoid heterogeneity resulting from unequal sampling, diversity indices were calculated at a sequencing depth corresponding to the read count of the sample containing the smallest set of sequences. Rarefaction plots and alpha diversity indices including chao1, shannon, simpson, goods coverage and observed species were calculated using the core_diversity_analyses.py script within the qiime pipeline (Caporaso *et al*., 2010b). For a beta diversity analysis, weighted and unweighted UniFrac phylogenetic distance matrices were constructed using the OTU table and the phylogenetic tree as input. Principal coordinate analysis (PCoA) plots were used to visualize phylogenetic relationships (Lozupone and Knight, 2005).

### Quantitative real‐time PCR analysis (RT‐PCR)

To monitor population dynamics of some major LAB contributing in silage fermentation process, a quantitative PCR analysis was performed on same DNA extracts used for amplicon‐based 16S rDNA sequencing. The species included were *Lactobacillus reuteri*,* Lactobacillus acidophilus*,* Lactobacillus brevis*,* Lactobacillus buchneri*,* Lactobacillus plantarum* and *Pediococcus pentosaceus*. Primers targeting intergenic spacer region of 16S‐23S rRNA genes were used for quantification of *L. acidophilus* and *L. reuteri* as described previously (Haarman and Knol, 2006). Primers previously designed to target *recA* gene were used for species‐specific quantification of *L. brevis*,* L. buchneri*,* L. plantarum* and *P*. *pentosaceus* (Stevenson *et al*., 2006). The sequence of primers used in this study is presented in Table S2. Each primer pair was first used to amplify the corresponding target amplicon using either genomic DNA of the isolated strain (for *L. Plantarum* and *L. buchneri*) or pooled DNA extract of all silage samples as template. PCR was run in a 25 μl reaction containing 2.2 μl of 25 mM MgCl_2_, 2.5 μl of 10 ×  reaction buffer, 2.0 μl of dNTP mix (10 mM), 1 μl of each primer (10 μM), 0.5 μl of Taq DNA polymerase (5 U μl^−1^), 2.0 μl of template DNA (20–30 ng μl^−1^ of genomic DNA) and 13.8 μl of nuclease‐free water. The resulting PCR product was gel‐purified using Qiaquick^®^Gel Extraction kit (Qiagen). The gel‐purified DNA fragments were TA‐cloned into the pGEM‐T Easy vector using the pGEM‐T Easy cloning kit (Promega, Madison, Wisconsin, USA) following the manufacturer's instruction. Plasmid contacting the target amplicon was quantified fluorometrically and used in 10‐fold serial dilution (1 × 10^8^ to 1 × 10^1^) for the generation of standard curve suitable for absolute quantification of the corresponding species. RT‐PCR was performed by a BioFACT™ 2X Real‐Time PCR SYBR Green Master Mix using the MyiQ™ single colour Real‐Time PCR Detection System (Bio‐Rad, Hercules, CA, USA). Standards and samples were assayed in a 20 μl of reaction mixture containing 10 μl of master mix, 1 μl of each primer (10 μM), 7 μl of nuclease‐free water and 1 μl of DNA template (2 ng). The amplification program was 95°C for 15 min, 40 cycles at 95°C for 10 s, 59–64°C for 20 s and 72°C for 20 s and a final extension at 72°C for 5 min. For each species, standards and samples were run on the same plate in triplicate. All standard curves were carefully curated to meet the required standard of efficiency (95% < efficiency < 115% and *R* ^2^ > 0.99). To determine the specificity of amplification, the melting curve of each amplicon was generated by slow heating from 60 to 95°C (alternating 0.5°C increments held for 5 s), with fluorescence collection at 0.5°C intervals.

### Metagenome functional prediction based on 16S rDNA sequence data

The functional potential of microbial community of silage samples prepared from maize biomasses grown in three contrasting sites was inferred via Phylogenetic Investigation of Communities by Reconstruction of Unobserved States (PICRUSt; Langille *et al*., 2013). For this analysis, quality filtered sequences were subjected to closed‐reference OTU picking based on 97% sequence similarity using greengenes OTUs (v. 13.5) as a template. The OTU table was normalized for 16S rRNA gene copy number using normalize_by_copy_number.py script implemented in the picrust software. The final metagenome prediction was performed using predict_metagenomes.py script. The metagenome table was then collapsed at level 3 of functional hierarchy and subjected to statistical analysis using the STAMP statistical package (Parks *et al*., 2014). One‐way analysis of variance (one‐way ANOVA) and Tukey's honest significant *post hoc* test were used to identify Kyoto Encyclopedia Genes and Genomes (KEGG) pathways and categories differed in abundance in the ensiled material between the three sampling sites and six storage time points.

### Statistical analysis

The data were subjected to analyses of variance using the General Linear Model procedure implemented in sas v9.2 software (SAS institute, Cary, NC, USA). Statistically significant differences between means were calculated using Duncan's multiple range test. To identify OTUs whose abundance differed significantly between the three sampling sites, an analysis of variance was conducted, in which the *P*‐value was adjusted by the Bonferroni correction. A spearman correlation analysis was performed between absolute abundance of species obtained by RT‐PCR and some physico‐chemical properties of silages using the observation_metadata_correlation.py script implemented in the Qiime pipeline. Differences were considered to be statistically significant when the *P*‐value was < 0.05.

## Conflict of interest

None declared.

## Supporting information


**Fig. S1**. Organic matter (OM) content of maize silage produced from biomass grown at three contrasting sites. The silages were sampled at 0, 3, 6, 14, 21 and 32 days post‐ensilage. Data are based on a dry matter content basis.
**Fig. S2.** Rarefaction analysis. The curves plot the number of detected OTUs as a function of the number of sampled reads.
**Fig. S3.** Shift in population of microbes constituting the epiphytic community of maize biomass before and after ensiling. Graph shows changes in the relative abundance (per cent of read assigned) of OTUs affiliated to four major microbial families (Lactobacillaceae, Leuconostocaceae, Acetobacteraceae and Enterobacteriaceae) present in the epiphytic community (day 0). The population dynamics of these families were also monitored after 3, 6, 14, 21 and 32 days post‐ensilage.
**Fig. S4.** Metagenome functional prediction using PICRUSt. Box whisker plots show the enrichment status of pathways displayed statistically significant differences between silages obtained from maize biomass grown in three contrasting sites. One‐way ANOVA and Tukey's post hoc test were used to identify differentially represented orthologs. A *P*‐value < 0.05 was considered statistically significant.
**Table S1.** the number of sequences generated per sample before and after quality filtering along with the number of OTUs detected before and after chimera filtering and OTU abundance filtering is presented.
**Table S2.** the sequence of primes used for real‐time PCR quantification of LAB species playing important role in maize silage fermentation.
