# Supplementary material for: The dynamics of the bacterial communities developed in maize silage
Source: Microb Biotechnol. 2017 Jul 11;10(6):1663–76. doi: 10.1111/1751-7915.12751 (PMC5658587; doi:10.1111/1751-7915.12751)
Supplement: Supplementary file 1 — Fig. S1. Organic matter (OM) content of maize silage produced from biomass grown at three contrasting sites. The silages were sampled at 0, 3, 6, 14, 21 and 32 days post‐ensilage. Data are based on a dry matter content basis. Fig. S2. Rarefaction analysis. The curves plot the number of detected OTUs as a function of the number of sampled reads. Fig. S3. Shift in population of microbes constituting the epiphytic community of maize biomass before and after ensiling. Graph shows changes in the relative abundance (per cent of read assigned) of OTUs affiliated to four major microbial families (Lactobacillaceae, Leuconostocaceae, Acetobacteraceae and Enterobacteriaceae) present in the epiphytic community (day 0). The population dynamics of these families were also monitored after 3, 6, 14, 21 and 32 days post‐ensilage. Fig. S4. Metagenome functional prediction using PICRUSt. Box whisker plots show the enrichment status of pathways displayed statistically significant differences between silages obtained from maize biomass grown in three contrasting sites. One‐way ANOVA and Tukey's post hoc test were used to identify differentially represented orthologs. A P‐value < 0.05 was considered statistically significant. Table S1. the number of sequences generated per sample before and after quality filtering along with the number of OTUs detected before and after chimera filtering and OTU abundance filtering is presented. Table S2. the sequence of primes used for real‐time PCR quantification of LAB species playing important role in maize silage fermentation. [file MBT2-10-1663-s001.docx]

Supplementary table 1: the number of sequences generated per sample before and after quality filtering along with the number of OTUs detected before and after chimera filtering and OTU abundance filtering are presented.

| SampleID | Number of raw reads | Number of reads passed split_libraries.py | Number of *de novo* OTUs | Number of OTUs after filtering for chimeric OTUs | Number of OTUs after filtering for low abundant  OTUs |
| --- | --- | --- | --- | --- | --- |
| Gorgan_0 | 18747 | 11877 | 540 | 493 | 151 |
| Gorgan_3 | 12891 | 8996 | 1085 | 1057 | 339 |
| Gorgan_6 | 10726 | 7770 | 1096 | 1081 | 352 |
| Gorgan_14 | 8499 | 5664 | 1038 | 1025 | 378 |
| Gorgan_21 | 7620 | 4970 | 875 | 869 | 348 |
| Gorgan_32 | 7113 | 4686 | 823 | 818 | 307 |
| Isfahan_0 | 7023 | 4761 | 210 | 200 | 91 |
| Isfahan_3 | 5741 | 3827 | 552 | 529 | 209 |
| Isfahan_6 | 4853 | 3153 | 475 | 459 | 196 |
| Isfahan_14 | 3535 | 2324 | 344 | 342 | 186 |
| Isfahan_21 | 3479 | 2382 | 343 | 343 | 191 |
| Isfahan_32 | 3407 | 2255 | 349 | 347 | 190 |
| Qazvin_0 | 12011 | 7479 | 1148 | 1042 | 301 |
| Qazvin_3 | 8484 | 5579 | 834 | 834 | 354 |
| Qazvin_6 | 6356 | 4196 | 737 | 736 | 320 |
| Qazvin_14 | 7324 | 4539 | 739 | 732 | 322 |
| Qazvin_21 | 6393 | 4110 | 760 | 757 | 318 |
| Qazvin_32 | 9203 | 4695 | 737 | 719 | 212 |
| Total | 143405 | 93263 | 6459 | 6232 | 842 |

Supplementary table 2: the sequence of primes used for real-time PCR quantification of LAB species playing important role in maize silage fermentation.

| Primer name | Primer sequence | Species | Target gene | Product size | TM  (ºC) |
| --- | --- | --- | --- | --- | --- |
| LacReu-F | ACCGAGAACACCGCGTTATTT | *L. reuteri* | 16S-23S | 93bp | 59 |
| LacReu-R | CATAACTTAACCTAAACAATCAAAGATTGTCT |  |  |  |  |
| LacAci-F | GAA AGA GCC CAA ACC AAG TGA TT | *L. acidophilus* | 16S-23S | 85bp | 59 |
| LacAci-R | CTTCCCAGATAATTCAACTATCGCTTA |  |  |  |  |
| LacBre-F | GCAGTTGCCGAGGTCCAA | *L. brevis* | recA | 64bp | 60 |
| LacBre-R | CCAACGCATTTTCAGCATCA |  |  |  |  |
| LacBuc-F | GGACCAATGCAGCAACTGAA | *L. buchneri* | recA | 72bp | 61 |
| LacBuc-R | AGATTACTGACGCATTGGTTACCA |  |  |  |  |
| LacPla-F | AGGCGCGGCTGATGTCA | *L. plantarum* | recA | 68bp | 60 |
| LacPla-R | CGCGATTGTCTTGGTTTTGTT |  |  |  |  |
| PedPen-F | CTATTGACTTGGTCGTTATTGATTCC | *P. pentosaceus* | recA | 72bp | 59 |
| PedPen-R | CCCCCATCTCTCCATCAATTT |  |  |  |  |
